# Laparoscopic Choledochotomy in a Solitary Common Duct Stone: A Prospective Study

**DOI:** 10.1155/2018/8080625

**Published:** 2018-05-14

**Authors:** K. B. Deo, S. Adhikary, S. Khaniya, V. C. Shakya, C. S. Agrawal

**Affiliations:** Department of Surgery, B. P. Koirala Institute of Health Sciences, Dharan, Nepal

## Abstract

**Background:**

Laparoscopic common bile duct exploration has all the advantages of minimal access and is also the most cost effective compared to the other options.

**Objective:**

To study a profile on laparoscopic common bile duct exploration for a single common duct stone.

**Methods:**

A total of 30 consecutive patients with solitary common bile duct stone attending our hospital over a period of one year were enrolled in the study. Laparoscopic common bile duct exploration was done by transductal route in all the patients.

**Results:**

There were 18 females and 12 males with age ranging from 28 to 75 years. Jaundice was present in 12 (40%) patients. Twenty-four (80%) patients had raised alkaline phosphatase. The mean size of CBD on ultrasound was 11.55 mm. The mean size of calculus was 11.06 mm and was located in the distal CBD in 26 (86.7%) patients. The mean operative time was 158.4 ± 57.89 min. There were 8 (26.6%) conversions to open procedure. T-tube was used in 26 (86.7%) patients. The postoperative complications were hospital acquired chest infection in 3 (10%), surgical site infection in 3 (10%), acute coronary syndrome in one (3.3%), and bile leak after T-tube removal in one (3.3%) patient.

**Conclusions:**

Laparoscopic common bile duct exploration is an effective, safe management of common bile duct stone.

## 1. Introduction

Approximately 10% of patients who undergo laparoscopic cholecystectomy harbor common bile duct stones [1, 2]. With continual improvement in the technology and expertise in laparoscopic techniques, laparoscopic common bile duct exploration is becoming more popular and may be the next paradigm in the management of choledocholithiasis [3]. Clearance rates of more than 90% are accepted as the standard of care [4].

During the early days of laparoscopic cholecystectomy, the use of intraoperative laparoscopic common bile duct exploration (LCBDE) was limited. Surgeons relied on methods like ERCP ± sphincterotomy with morbidity (15%) and mortality (1%), which increased the hospital stay and cost with additional risk of acute pancreatitis [5]. This has led to resurgence of LCBDE in common duct stones [5]. We conducted this study to find out the safety of LCBDE in our patients.

## 2. Materials and Methods

It is a prospective study in patients with solitary duct stones with or without jaundice. Multiple duct stones, CBD diameter < 6 mm, cholangitis or pancreatitis, previous history of cholecystectomy, dense and ugly abdominal scars, and those unwilling to undergo surgery and unfit for surgery were excluded.

A good history and clinical examination followed by routine work-up and ultrasonography was done to see the gall stones, number, size, and location of common duct stones, and diameter of common duct. The diagnosis of single choledocholithiasis was made only after sonologist confirmed visualizing entire common duct in one or more than one setting.

Laparoscopic CBD exploration was done with 10 mm umbilical and epigastric ports followed by two small accessory subcostal ports. The first 5 mm trocar was placed along the right anterior axillary line and second 5 mm port at the right subcostal region. An additional right epigastric 5 mm port was useful for inserting a rigid ureteroscope. The choledochotomy was closed with 3-0 polyglactin acid over the T-tube. The T tubes were made up of polyvinyl chloride. A subhepatic closed suction drainage (14 F) was then inserted. Visual analogue scale scoring was done in the postoperative period for assessment of pain. T-tube cholangiogram was done on 10th day to look for any retained stones or abnormal findings. T-tube was removed only after confirmation of normal findings in cholangiogram. All the data were entered in Microsoft excel and converted to SPSS version 11.5.

## 3. Results

Thirty subjects of LCBDE included 14 (46.7%) cases in the age group between 40 and 60 years. The mean age was 49.2 ± 12.89 years with a male to female ratio of 1.5 : 1 (Table 1).

All cases had abdomen pain and duration varied from 15 days to 9 months with a mean of 94.7 days. Jaundice was seen in 12 (40%) patients with mean duration of 16.25 days. Eight patients had comorbid conditions, namely, hypertension (4), chronic obstructive pulmonary disease (2), and type II diabetes mellitus and hypertension (2), and two cases had abdominal hysterectomy in the past. On physical examination, icterus was present in 12 (40.0%) patients, pallor in two (6.6%) patients, and hepatomegaly in two (6.6%) patients. Twenty-four (80%) patients had deranged liver function test. Total bilirubin was raised in 12 (40.0%) and alkaline phosphatase was raised in 24 (80.0%) patients. The prothrombin time and international normalized ratio (INR) were raised in 11 (36.7%) patients.

Abdominal ultrasonography was the main tool for diagnosis as our institution does not have magnetic resonance cholangiography (MRC) facility. All had associated gallstones. The most common location of stone was at the distal common duct 26 (86.7%) and in four (6.6%) cases it was in the mid part of duct. The mean diameter was 11.55 ± 2.43 mm (range 8 to 17 mm); the mean size of the stone was 11.06 ± 4.42 mm (range 5.7 to 25.0 mm).

The mean operative time for laparoscopic surgery was 158.40 ± 57.89 minutes (range 75 to 360 mins) (Table 1). In those who had to be converted, one patient had a prolonged operative interval (360 mins) because we performed Roux en Y hepaticojejunostomy for choledochal cyst.

**Figure 1:**
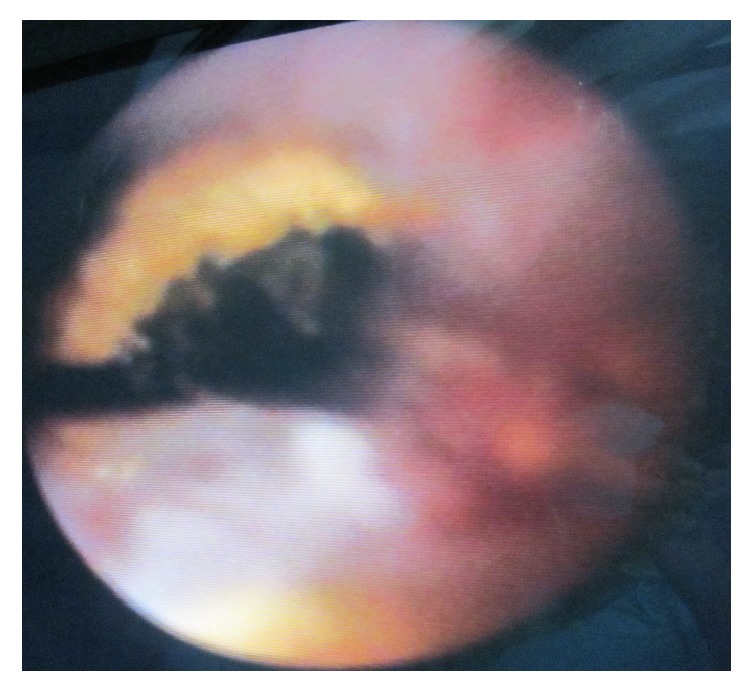


**Figure 2:**
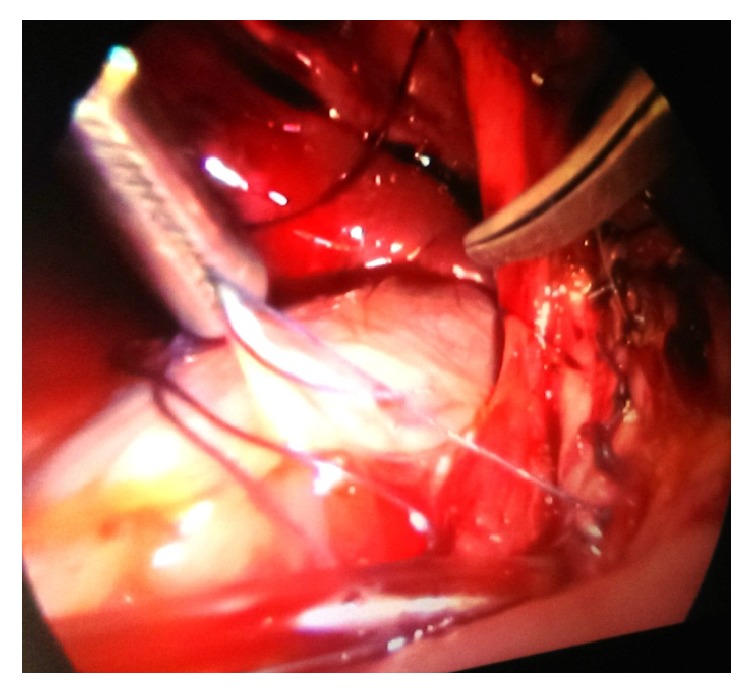


Laparoscopic exploration was done successfully (Figures 1 and 2) in 22 (73.3%) cases and 8 (26.6%) had to be converted (Table 1). The reasons for conversion were: multiple stones (1), impacted calculus in the distal end of duct which was difficult to extract (2), frozen anatomy of Calot's triangle (1), nonretrieval of a stone (1), and requirement of choledochoduodenostomy (2). One patient had to undergo a Roux en y hepaticojejunostomy after excision of choledochal cyst (accidental discovery, not picked up by imaging). There were no intraoperative complications except bleeding from one branch of common hepatic artery to CBD (around 50 ml) which was controlled by application of pressure followed by electrocoagulation.

The postoperative period was fairly stable. The mean duration of analgesics administered was 5.9 ± 1.51 days (range 4 to 11 days). The visual analogue scale was used to assess the pain on days 1 and 2. The mean VAS score on the first postoperative day was 6.6 ± 1 (range 5 to 8 days). Similarly, the mean VAS score on the second day was 3.33 ± 0.76 (range 2 to 5 days).

Majority, 26 (86.7%), of patients recovered from ileus on the first day. Feeding was started on the second day for 24 (80%) patients. The mean complete ambulation time was 4.33 ± 1.49 days (range 3 to 10 days). Two patients had delayed ambulation due to cardiac (1) and chest complications (1). The mean hospital stay was 6.76 ± 1.33 days (range 5 to 11 days).

T-tube was used in 26 (86.7%) patients. One patient underwent primary closure after choledochotomy and the remaining two underwent choledochoduodenostomy as CBD diameter was more than 15 mm. Remaining one had Roux en Y hepaticojejunostomy after excision of choledochal cyst. Twenty-four had normal findings with free flow of contrast to the duodenum. Two patients had filling defect which was seen in the common bile duct. The T-tube removal was done before 21 days in 19 patients but all after 15 days. T-tube was removed after 21 days in 7 patients.

Three (10%) patients had developed hospital acquired chest infection. Surgical site infection (SSI) was present in three (10%) patients (two in conversion and one had port site infection following laparoscopic drainage of pyoperitoneum following bile leak after T-tube removal). SSI were managed with dressings and antibiotics. One patient developed acute coronary syndrome on the first day and he recovered with no further complication and was successfully discharged. Two patients with filling defect underwent endoscopic retrograde cholangiography and stone extraction for residual stones. One case had bile leak followed by pyoperitoneum following T-tube removal (at the end of 3rd week) and was subsequently managed with laparoscopic peritoneal lavage and drainage after readmission. She recovered gradually and had an incisional hernia in the umbilical port following a port site infection. The hernia was later managed with overlay mesh.

## 4. Discussion

Common bile duct stones are commonly managed by ERCP ± sphincterotomy, followed by laparoscopic cholecystectomy. The risks associated with ERCP and the morbidity associated with open surgery have paved the way for considering Laparoscopic common bile duct exploration [5]. Recently a single stage Laparoscopic common bile duct exploration has been increasingly reported as a safe and effective treatment option [6].

It is a prospective study of 30 consecutive patients of solitary common bile duct stone showing a higher prevalence in females (60.0%) comparable to other studies [7–10], which could be due to female gender, hormones, and increased fat consumption with less physical work. The mean age was 49.2 ± 12.89 years with majority between 40 and 60 years of age similar to other studies [11–13]; their mean age varied from 42.25 to 47 years; but in some other studies [8, 9, 14] they had presented in older age (mean age 63 to 66.1 years). In our study the mean age of presentation was 56.0 years in males and 44.66 years in females. This shows that common bile duct stone occurs early in females. The importance of age, as it affects a postoperative outcome, was shown in the study by Noble et al. [15] where patients beyond 60 years were more likely to suffer from respiratory and urinary complications.

The pain was present in all patients and it varied from 15 days to 9 months. Jaundice was present in 12 (40.0%) patients with a mean of 16.25 days and fever in 5 (16.7%) patients. Riciardi et al. [7] and Shelat et al. [10] also showed that abdomen pain was the major complaint which was present in 93% and 80% of the total cases, respectively. The presence of jaundice was similar in a study by Shelat et al. [10] but less (27%) in study by Riciardi et al. [7] possibly due to increased concerns shown by patients once jaundice appeared and also a small sample size of our study, and so on. Similarly fever was present in 14.7% and 15% in the study done by Riciardi et al. [7] and Rogers et al. [12], respectively, which was similar to our study. The late presentation seen in our few patients was because of difficult access to hospital.

Alkaline phosphatase was raised in 24 (80.0%) patients with a median of 253 U/L, higher than that in the study by Ricardi et al. [7] where alkaline phosphatase was raised only in 56% of the patients with a mean value of 216 ± 10 U/L. Similar findings were noticed by Rogers et al. [12] and Wani et al. [13].

Ultrasonogram of the abdomen was the main tool to diagnose common bile duct stone as magnetic resonance cholangiopancreaticography (MRCP) was not available at our institute and many of our patients cannot afford the cost of MRCP outside. The decision to operate was made only after sonologist had confirmed having visualized entire common duct and single stone in one or more setting. The most common location of the stone was distal CBD with as many as 26 (86.7%) patients with a mean diameter of 11.5 ± 2.43 mm (range 8 to 17 mm) comparable to a study by Chander et al. [11] where the average diameter was 11.7 mm and majority (63%) of patients had the diameter between 8 mm and 15.4 mm and Topal et al. [9] where the average diameter was 11.5 mm. However, Wani et al. [13] and Khan et al. [16] studies showed the mean common bile duct of 15 mm diameter. We chose to have solitary calculus as inclusion criteria as we were in the beginning of learning curve.

We used rigid ureteroscope for the visualization of common bile duct stone. Khan et al. [16] had also reported a successful use of a rigid nephroscope for laparoscopic common bile duct explorations. LCBDE was successfully done in 22 (73.3%) patients. The conversion was needed in 8 (26.6%) patients for impacted stones, frozen anatomy due to dense adhesions, multiple stones and associated technical difficulties, and nonretrieval of a stone. The finding of multiple stones in one case was in intraoperative period which was missed in imaging. Conversion rate was high in our study than other studies [7–10, 17–21] where it varied between no conversions in Bandyopadhaya et al. [18] study to 4% in others [10, 20]. The reasons for conversion in their studies were learning curve, dense adhesions, bleeding, technical difficulties, impacted stones, and so on. These reasons were similar to our study. Similar to our study choledochal cyst was one of the causes for conversion in study by Petelin [17] and Paganini and Lezoche [19] where two cases had bile duct cyst. The high conversion in our study could be due to a steep learning curve, being in early part of the study (initial thirty cases), the operative findings, technical difficulties, and so on. Due to the initial part of our learning curve we chose to convert two cases requiring choledochoduodenostomy due to hugely dilated CBD of almost two centimeters (we usually perform choledochoduodenostomy in case of CBD dilatation > 1.5 cm). The decision to perform choledochotomy was purely due to preoperative and intraoperative findings as we do not have facilities like laparoscopic ultrasound or intraoperative cholangiogram. However none of the patients had negative choledochotomy.

In our study the mean operative time was 158.4 ± 57.89 minutes (range 75 to 360 minutes). Our operative time was comparable with some studies [7, 11, 19] where mean operative time varied from 126 minutes to 139 minutes. Shorter operative time was seen in other studies [16, 18, 21] (range 71 mins to 83 mins). Some studies [10, 22] had a longer operative time (185 to 191 mins).

We started feeding on 2nd day and ambulation on the third day. However in study by Bandyopadhyay et al. [18] patients were started orally on the day of surgery and were ambulatory next day. The mean hospital stay was 6.76 ± 1.33 days ranging from 5 to 11 days. The hospital stay in our study was longer than other studies [7, 10, 11, 16, 18, 21, 23] where mean hospital stay varied from 1.95 days to 4.6 days. The mean hospital stay was longer in study by Tang et al. [14] and Huang et al. [22] (range 9 and 10.4 days). Longer hospital stay in our study was because of complications like chest infections, surgical site infections, and acute coronary syndrome.

The choledochotomy was closed over T-tube in majority (86.7%) of our patients. The decision of primary closure in one case was as per decision of operating surgeon. T-tube was used in all laparoscopic common bile duct explorations by Huang et al. [22]. However in some studies [7, 8, 13, 14, 16, 18] T-tube had been used in limited number of patients. They did primary closure of choledochotomy or used antegrade biliary stents. Wani et al. [13] had shown successful use of endonasobiliary drainage. Shimizu et al. [24] have described the use of the C tube through the cystic duct with a better outcome in terms of technical feasibility, lesser complication, early removal, and decreased hospital stay as compared to the use of T-tube. In our study average removal time was 20.92 ± 8.0 days (range 15 to 42 days). This was quite long as compared to a study by Chander et al. [11] where the average removal time was 13.1 ± 5 days. The reason for variation in the removal of T-tube in our study was the removal on an outpatient basis as some patients presented late, as well as presence of filling defects in CBD in two (6.6%) patients where we had to continue with the tube till a definitive treatment was done. Similarly other studies also had retention of stones (2 to 8%) in their patients [7, 11, 14, 19, 22].

The postoperative complications were similar to other studies [7, 8, 11, 14, 16, 19, 21] which include bile leak, chest and port site infection, and myocardial infarction. Bile leak leading to pyoperitoneum was present in one of our patients following T-tube removal at the end of 3rd week. It was unusual compared to our past experience with PVC T-tube. Though formation of fibrous tract is slow as compared to latex tube, 3 weeks used to be a sufficient time. Though this study did not have mortality, it was present (1 to 3%) in other studies [14, 19, 21]. It could be due to selection bias, a small sample size, and less patients with comorbid condition. The technique has been reported to be associated with morbidity and mortality rates that range as high as 5% to 7% and 1% to 2%, respectively [25].

## 5. Limitations of Our Study

This is a prospective study with a small sample size. Moreover, it was our initial experience that there could have been a little selection bias. We look to conduct future studies with a much bigger sample size and conduct a prospective randomized controlled trial comparing it with other options available. We had to deviate from established methods of investigations like MRCP and few intraoperative steps (like choosing only transductal approach over transcystic approach, using intraoperative cholangiogram or laparoscopic ultrasound) due to unavailability and cost factor in our patients.

## 6. Conclusion

In the era of minimal access surgeries, laparoscopic common bile duct exploration is an effective, safe management of common bile duct stone. Besides advantages of laparoscopic surgery, it offers a single stage management of common bile duct stone reducing the hospital stay and financial load to the patients.

## Figures and Tables

**Table 1:** 

Parameters	Numbers (*n* = 30)
Age group in years	
<40	9
40–60	14
≥60	7
Gender	
Male	12
Female	18

Symptoms	
Abdomen pain	30
Jaundice	12
Fever	5

Mean operative time	158.40 ± 57.89 (75–360) min

Total conversions	8 (26.7%)

Reasons for conversion	
Impaction	2
Frozen anatomy	1
Multiple stones	1
Nonretrieval of stone	1
Choledochoduodenostomy	2
Hepaticojejunostomy (choledochal cyst)	1

Complications	
Morbidity	7 (23.3%)
Chest infection	3
Surgical site infection	3
Acute coronary syndrome	1
Mortality	0
